# Chronic Mercury Toxicity Associated With Habitual Dried Shark Consumption in Oman: A Case Report

**DOI:** 10.7759/cureus.116693

**Published:** 2026-09-21

**Authors:** Fatma Al Kharusi, Redha Al Lawati, Badria Alhatali

**Affiliations:** 1 Emergency Medicine, Oman Medical Specialty Board, Muscat, OMN; 2 Internal Medicine, Royal Hospital, Muscat, OMN; 3 Respiratory Medicine, Canberra Hospital, Canberra, AUS; 4 Toxicology, National Poison Centre Oman, Muscat, OMN

**Keywords:** dried shark, fish consumption, heavy metal poisoning, mercury toxicity, methylmercury, oman, seafood exposure

## Abstract

Mercury exposure is closely linked to the consumption of contaminated fish, particularly those containing methylmercury. Mercury toxicity presents with a wide range of nonspecific symptoms, making diagnosis challenging for clinicians. The clinical manifestations vary depending on the type of mercury, levels and duration of exposure, and individual susceptibility.

We report a case of a 69-year-old woman with no significant past medical history who presented to the Emergency Department (ED) with chronic abdominal pain, muscle aches, bloating, and generalized fatigue lasting over a year. An initial evaluation in Thailand revealed an elevated blood mercury level of 38.72 μg/L during evaluation in Thailand. She was offered chelation therapy but declined and sought a second opinion in Oman. Repeated testing at the Central Public Health Laboratories two months later showed a mercury level of 24.36 μg/L (reference range <15 μg/L). Her complete blood count, renal function, and liver function tests were within normal limits. Dietary history revealed daily consumption of seafood and dried fish, including shark, as her primary protein source. Following cessation of dried fish consumption and reduction of seafood intake, blood mercury levels normalized without chelation therapy. Seafood, particularly dried fish, can be a significant source of methylmercury exposure.

This case emphasizes the importance of obtaining a detailed dietary history when evaluating patients with chronic, nonspecific symptoms. Identifying dried fish as a key source of mercury exposure in Oman highlights the need for public awareness and early recognition of environmental toxic exposures.

## Introduction

Mercury exists in elemental, organic, and inorganic forms, all of which are hazardous to human health. It is the only metal that remains in a liquid state at room temperature and exhibits lipophilic properties, enabling it to penetrate cell membranes and accumulate in various organ systems, including the central nervous system, endocrine system, urinary system, and gastrointestinal system. Mercury is widely used in several industries, such as agriculture, lighting, battery manufacturing, jewellery, dye production, and dentistry. The severity of mercury toxicity in humans depends on several factors such as the route of exposure, duration of contact, and individual susceptibility [1]. Seafood is a major dietary source of methylmercury, the most neurotoxic form of mercury [2]. However, the nonspecific nature of mercury poisoning symptoms often makes diagnosis particularly challenging for clinicians [3].

In Oman, fish is a staple component of the diet, especially in coastal regions where it serves as a primary source of protein. Recent studies have raised concerns about mercury accumulation in locally consumed species, especially dried fish, posing a potential environmental and public health risk.

We report this case to highlight the potential risk of mercury toxicity associated with habitual dried shark consumption and the importance of obtaining a detailed dietary history in patients presenting with nonspecific symptoms. To our knowledge, this is one of the first reported clinical cases of mercury toxicity associated with habitual dried shark consumption in Oman.

## Case presentation

A 69-year-old woman with no significant medical history presented to the Emergency Department (ED) following a routine check-up in Thailand one month earlier. During her evaluation in Thailand, she reported chronic muscle pain, generalized fatigue, and abdominal bloating that had been present for approximately one year. At the time of presentation to our ED one month later, the abdominal bloating and muscle pain had largely resolved, although generalized fatigue persisted.

In Thailand, general laboratory screening revealed an elevated whole blood mercury level of 38.72 μg/L; however, the reference range was not reported. She was offered chelation therapy but declined, opting instead to seek a second opinion in Oman.

Dietary history revealed a daily intake of seafood, including dried shark, shrimp, and fresh tuna, which constituted the main sources of protein in her diet. On physical examination, the vital signs were normal. Head and neck, respiratory, cardiovascular, and abdominal examinations were unremarkable. Neurological examination revealed normal muscle strength, tone, reflexes, coordination, sensation, and gait. There was no evidence of tremor, paraesthesia, ataxia, spasticity, or other focal neurological deficits, and there was no hearing or vision loss. No neuropsychiatric abnormalities were observed.

Her whole blood mercury level at presentation was 24.36 μg/L (reference range < 15 μg/L), and her arsenic level was 3.4 μg/L. She was advised to reduce seafood consumption to a maximum of three times per week and return for follow-up testing after one month.

Upon follow-up 35 days later, she reported adherence to the dietary recommendations, including complete cessation of dried shark consumption and limiting seafood intake to no more than three times per week. Repeated testing showed a reduction in whole blood mercury level to 11.88 μg/L and urinary mercury level to 0.82 μg/L (Table 1). The patient reported marked improvement in energy levels and reduction in muscle aches following dietary modification. Abdominal bloating, which had resolved prior to presentation in Oman, did not recur.

**Table 1:** Blood and Urinary Mercury Levels Before and After Dietary Modification Laboratory investigations over time. Day 1 represents the initial assessment in Thailand; Day 26 represents the presentation in Oman; Day 61 represents the follow-up assessment after dietary modification.

Date	Investigation	Result
Day 1	Whole blood mercury level	38.72 μg/L (reference range <15 μg/L)
	Arsenic level	10.8 μg/L (reference range <10 μg/L)
Day 26	Whole blood mercury level	24.36 μg/L (reference range <15 μg/L)
	Complete blood count	Within normal limits
	Renal function tests	Within normal limits
	Liver function tests	Within normal limits
	Bone profile	Within normal limits
	Whole blood lead level	1.2 μg/dL (reference range <30 μg/dL)
	Arsenic level	3.4 μg/L (reference range <10 μg/L)
Day 61	Whole blood mercury level	11.88 μg/L (reference range <15 μg/L)
	Urinary mercury level	0.82 μg/L (reference range <10 μg/L)

Ethical approval was granted by the Ethics Committee of the Royal Hospital with the number MOH/CSR/CR/26/16, dated April 30, 2026.

## Discussion

Mercury is a highly toxic, non-essential heavy metal that exists in both organic and inorganic forms. Inorganic mercury includes elemental mercury and mercuric salts, both of which are toxic, though their absorption and clinical manifestations differ. The severity of poisoning depends on various factors, such as the type, intensity, and duration of exposure [1,2]. Organic mercury, particularly methylmercury and dimethylmercury, is the most prevalent and hazardous form encountered in environmental exposure, especially through seafood consumption [3]. Methylmercury bioaccumulates in aquatic food chains, particularly in swordfish, sharks, and tuna. Elemental mercury is primarily absorbed through the lungs, and ionic mercury salts have different absorption rates through the gastrointestinal tract and lungs, with minimal absorption through the skin [4].

Common sources of mercury exposure include barometers, batteries, bronzing agents, calibration instruments, dental amalgams, electroplating, the jewellery industry, paints, silver and gold production, and thermometers. Seafood, especially contaminated shellfish and dried fish, is a significant source of mercury. Methylmercury, the most toxic form of mercury, is primarily acquired through the consumption of contaminated fish and has the most detrimental effect on brain development. Methylmercury has a long biological half-life and is barely eliminated by cooking. Studies have shown that prenatal exposure to mercury through seafood in the maternal diet can increase the risk of neurodevelopmental issues in children.

Acute inhalation of elemental mercury can cause symptoms such as fever, chills, dyspnoea, metallic taste, pleuritic chest pain, stomatitis, and lethargy. While recovery is often complete, pulmonary complications might occur [5]. Chronic mercury exposure can lead to renal failure, dementia, acrodynia, tremors, gingivitis, erythrism, tunnel vision, and ataxia. Moreover, due to a lack of understanding of mercury poisoning and its peripheral neuropathy symptoms, the condition is often misdiagnosed [6]. Clinicians should consider mercury poisoning in patients presenting with chronic, vague symptoms like memory loss, sleep disturbances, fatigue, pain, or other nonspecific signs, and promptly check blood and urine mercury levels [5,7].

In this case, the patient’s elevated blood mercury level 38.72 μg/L in Thailand; 24.36 μg/L in Oman) was likely attributed to her habitual consumption of dried shark and fresh tuna, species known to contain high concentrations of mercury. Dried shark is commonly consumed in Oman. Shellfish and dried shark are among the highest mercury- contaminated seafood, with mean reported mercury concentrations of 0.23 mg/kg, posing a significant risk to consumers [2]. Her symptoms, chronic fatigue, muscle pain, and abdominal bloating, were nonspecific but consistent with subacute mercury toxicity. Importantly, the patient’s neurological examination was normal, and she did not exhibit signs of neurotoxicity such as tremors, paraesthesia, or ataxia. This aligns with literature suggesting that clinical manifestations of mercury toxicity vary widely depending on dose, duration, and individual susceptibility. Chelation therapy is typically reserved for patients with severe symptoms or markedly elevated levels. In this case, conservative management through dietary modification alone led to a substantial decline in blood mercury levels to 11.88 μg/L and resolution of symptoms. 

A baseline urinary mercury measurement was not available, limiting assessment of trends over time and differentiation between organic and inorganic mercury exposure.

A mildly elevated arsenic level was noted during the initial assessment (10.8 µg/L; reference range <10 µg/L); however, the elevation was minimal and normalized on repeat testing. Given its marginal elevation and lack of clinical features suggestive of arsenic toxicity, it was not considered clinically significant in this case.

A limitation of this case is that a comprehensive assessment of non-dietary mercury exposure sources, including occupational, environmental, and household exposures, was not systematically documented. Although the patient's frequent consumption of dried shark and other seafood strongly suggested a dietary source of mercury exposure, alternative sources cannot be completely excluded.

As a single-patient case report, causality cannot be established with certainty, and unidentified confounding factors may have contributed to the clinical findings.

## Conclusions

This case demonstrates that habitual consumption of dried shark can result in clinically significant mercury exposure even in the absence of neurological manifestations. A detailed dietary history remains essential when evaluating patients with chronic nonspecific symptoms, particularly in coastal populations with high seafood consumption. This case also highlights the importance of public awareness regarding the potential risks associated with frequent consumption of high-mercury seafood, particularly dried shark.
